# The spiegelian hernia: a rare cause of acute intestinal obstruction

**DOI:** 10.11604/pamj.2015.20.113.5982

**Published:** 2015-02-09

**Authors:** Youssef Omor, Stephane Alard

**Affiliations:** 1 Department of Radiology, University Hospital of Saint-Pierre, Brussels, Belgium

**Keywords:** Spiegelian hernia, intestinal obstruction, abdominal CT

## Image in medicine

An 73-year-old man, with no abdominal surgical history, presented to the emergency in an array of acute intestinal obstruction. Abdominal CT-Scan axial (A) and coronal (B) sections were performed objectifying hernia sac containing intestinal structure hail externalized through a collar on the outer edge of the rectus muscle on Spiegel's line with no sign of digestive sufferance. The laparoscopic total extraperitoneal mesh repair of the Spiegel hernia was performed. The postoperative course was uneventful and the patient left the hospital on the seventh postoperative day after resumption of normal intestinal transit. Spiegelian hernia is a rare type of ventral abdominal hernia. It occurred in patients in the 6th and 7th decade of life, with an equal sex prevalence. The pathogenesis often involves a dehiscence of the transverse and internal oblique muscle aponevrosis. The diagnosis of Spiegelian hernia is difficult because no characteristic symptoms are identified and often there is no palpable mass, therefore only 50% of cases are diagnosed preoperatively. The abdominal CT scan is helpful in the description of hernia's topography and sometimes in diagnostic confirmation. Surgical treatment is recommended due to the high risk of complications like incarceration and strangulation.

**Figure 1:**
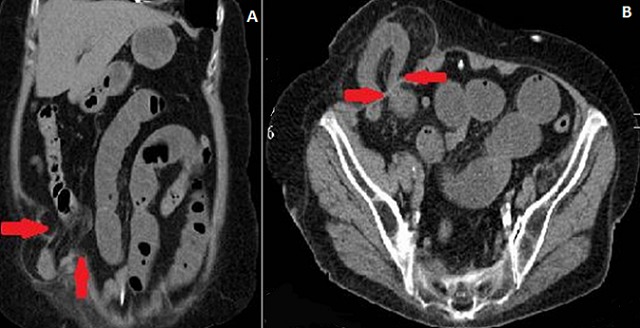
(A) abdominal CT-Scan axial and (B) coronal sections

